# Tamoxifen and clomiphene inhibit SARS-CoV-2 infection by suppressing viral entry

**DOI:** 10.1038/s41392-021-00853-4

**Published:** 2021-12-21

**Authors:** Shulong Zu, Dan Luo, Lili Li, Qing Ye, Rui-Ting Li, Yanan Wang, Meiling Gao, Heng Yang, Yong-Qiang Deng, Genhong Cheng

**Affiliations:** 1 Center for Systems Medicine, Institute of Basic Medical Sciences, Chinese Academy of Medical Sciences and Peking Union Medical College, Beijing, China; 2 Institute of Systems Medicine, Chinese Academy of Medical Sciences and Peking Union Medical College, Beijing, China; 3 Suzhou Institute of Systems Medicine, Suzhou, Jiangsu China; 4 State Key Laboratory of Pathogen and Biosecurity, Beijing Institute of Microbiology and Epidemiology, Academy of Military Medical Sciences, Beijing, China; 5 Suzhou Func Biotech Inc, Suzhou, Jiangsu China; 6 Department of Microbiology, Immunology and Molecular Genetics, University of California, Los Angeles, CA USA

**Keywords:** Drug discovery, Microbiology

**Dear Editor**,

COVID-19 pandemic caused by severe acute respiratory syndrome coronavirus 2 (SARS-CoV-2) is still a threat to millions of lives worldwide. Although SARS-CoV-2 vaccines have been approved to reduce the severity and death associated with COVID-19, the number of SARS-CoV-2-infected cases still remains high, especially with the appearance of various mutant strains such as P.1.351 and P.1.617 (also known as South Africa strain and India strain, respectively), which may reduce the efficacy of vaccine protection. There is an urgent need to develop effective antiviral agents to treat COVID-19 patients, especially with those infected with SARS-CoV-2 variants of concern.

The goal of our study was to evaluate the SARS-CoV-2 antiviral activities of Food and Drug Administration (FDA)-approved drugs, which could accelerate the development of novel therapies for COVID-19. We tested tamoxifen and clomiphene which were also used to screen antiviral agents against flavivirus. On Vero and Caco-2 cells, we first measured the cytotoxicity of the two drugs and determined that their median cytotoxic concentrations (CC_50_s) in vitro were much higher than our experimental range (Supplementary Fig. S1a–d). Moreover, we analyzed the antiviral activities of the two drugs and found that both tamoxifen and clomiphene had effective antiviral activities. The median effective concentrations (EC_50_s) of tamoxifen and clomiphene on Vero cells were 1.634 and 0.3213 μM, respectively (Fig. 1a). The two drugs also suppressed SARS-CoV-2 infection in Caco-2 cells with a dose-dependent manner (Supplementary Fig. S1e, f). These results showed that tamoxifen and clomiphene strongly antagonized SARS-CoV-2 infection in vitro.

**Fig. 1:**
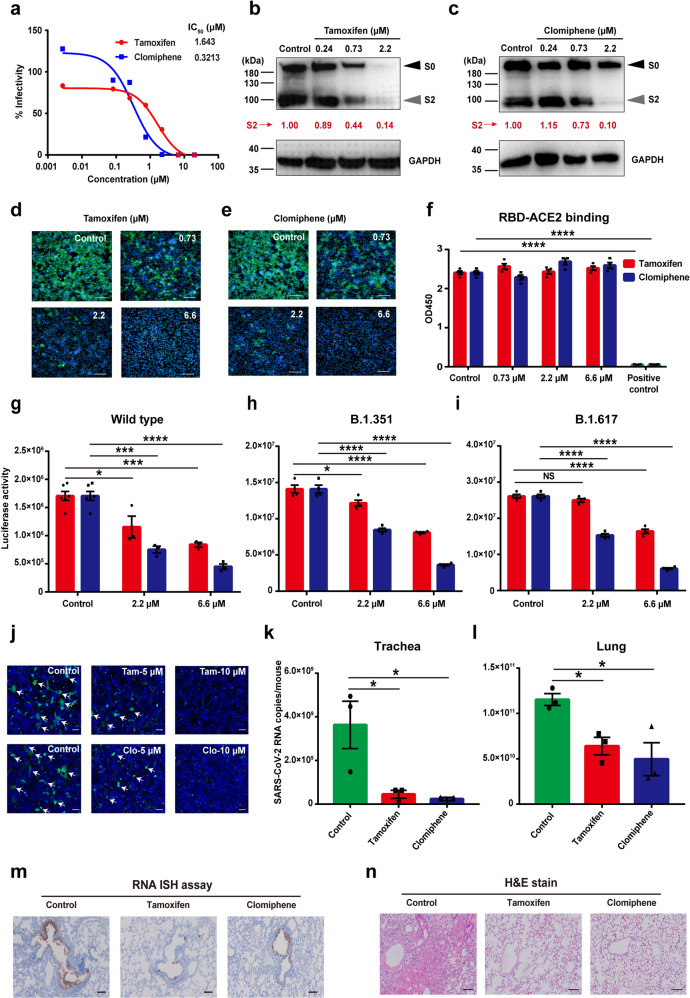
Tamoxifen and clomiphene inhibit SARS-CoV-2 infection in vitro and in vivo. **a** Vero cells were pretreated with different doses of tamoxifen or clomiphene for 12 h, then the cells were infected with SARS-CoV-2 at 100 median tissue culture infectious dose (TCID_50_). The cell supernatants were used to test virus RNA loads by qRT-PCR at 48 h post infection (hpi). The EC_50_s of tamoxifen and clomiphene on Vero cells were indicated. **b**, **c** Vero cells were pretreated and infected as in (**a**). After 48 h, the cells were collected and treated for western blot. SARS-CoV-2 S protein and GAPDH were tested. The black arrow indicates bands corresponding to uncleaved S proteins (S0) and gray arrow indicates bands corresponding to the S2 subunit. The quantitative results of the expression of S protein of SARS-CoV-2 were labeled. **d**, **e** Vero cells were pretreated and infected as in (**a**). After 48 h, the cells were fixed and treated for immunofluorescence assay. SARS-CoV-2 S protein was stained in green, and DAPI in blue. Scale bars, 100 μm. **f** RBD of SARS-CoV-2 S protein and ACE2 protein were used in ELISA assay to test the inhibition of tamoxifen and clomiphene to the binding activity. SARS-CoV-2 antibody was used as a positive control. **g** Huh7 cells were pretreated with tamoxifen and clomiphene, then were infected with SARS-CoV-2 pseudovirus. After 24 h, the cells were collected to examine the luciferase activity of pseudovirus. **h**, **i** Huh7 cells were treated as in (**g**), then were infected with P.1.351 (**h**) and P.1.617 (**i**) pseudovirus. After 24 h, the cells were collected to examine the luciferase activity of pseudovirus. **j** 293T cells were transfected with GFP-tagged SARS-CoV-2 S protein-expressing plasmid and then co-cultured with Huh7 cells. After 12 h, the cells were treated with tamoxifen and clomiphene for 24 h, and fixed with 4% paraformaldehyde, the membrane fusion was observed by fluorescence microscopy, white arrows indicate syncytium formation. Scale bars, 100 μm. **k**, **l** The mice trachea and lung were collected for qRT-PCR to test the virus loads. **m**, **n** Mice lungs were fixed with 4% paraformaldehyde for RNAscope to display SARS-CoV-2 RNA in mice lung (**m**) and stained with H&E to show the inflammatory cell infiltration (**n**). Scale bars, 100 μm. Quantitative data are represented as mean ± SEM; ns, not significant, *P ≤ 0.05; **P ≤ 0.01; ***P ≤ 0.001; ****P ≤ 0.0001

In addition to the EC_50_s calculated according to SARS-CoV-2 RNA copies in cell supernatants, we also measured the spike (S) protein levels of SARS-CoV-2 in infected cells under drug treatments. Vero cells were pretreated with tamoxifen or clomiphene of different doses, and then infected with SARS-CoV-2. Vero cells were continued to be treated with drugs after infection. SARS-CoV-2 S protein was used to test viral protein production by western blot. The results suggested that tamoxifen and clomiphene reduced SARS-CoV-2 S protein production, especially the accumulation of the S2 fragment (Fig. 1b, c). SARS-CoV-2 S protein was also examined under tamoxifen and clomiphene treatments by immunofluorescence (Fig. 1d, e). The results showed that the two drugs reduced SARS-CoV-2 protein production in a dose-dependent manner, which further illustrated the antiviral activities of tamoxifen and clomiphene against SARS-CoV-2 infection.

To understand the mechanism responsible for the antiviral activities of tamoxifen and clomiphene against SARS-CoV-2 infection, we first measured the transcriptome of Huh7 cells which were either untreated or pretreated with tamoxifen or clomiphene before SARS-CoV-2 infection to determine the effects of these drugs on SARS-CoV-2-induced gene expression profiles. The transcriptome results showed that SARS-CoV-2 infection increased the expressions of interferon (IFN) and acute inflammatory response genes, but these inductions were suppressed by tamoxifen and clomiphene treatments (Supplementary Fig. S2a, b). To further determine whether tamoxifen and clomiphene directly suppressed IFN and inflammation response genes or indirectly affected IFN and inflammation responses through inhibiting SARS-CoV-2 infection, we also analyzed the effect of tamoxifen and clomiphene on poly(I:C)-induced IFN response genes. A549 cells were pretreated with tamoxifen and clomiphene before transfection with poly(I:C) and cells were collected for real-time quantitative reverse transcription PCR (qRT-PCR). The results showed that tamoxifen and clomiphene did not affect the poly(I:C)-induced IFN response genes including *RIG-I*, *IFN-β,* and *IRF7* (Supplementary Fig. S3a–c), suggesting that tamoxifen and clomiphene control SARS-CoV-2 infection to reduce IFN and acute inflammatory responses.

To investigate the additional mechanisms by which tamoxifen and clomiphene inhibit SARS-CoV-2 infection, we applied the SARS-CoV-2 pseudovirus, which only contains the S protein of SARS-CoV-2 on the VSV backbone with a luciferase reporter. We found that tamoxifen and clomiphene significantly suppressed the infection of wide type (WT) SARS-CoV-2 pseudovirus in a dose-dependent manner (Fig. 1g). Moreover, we also found that tamoxifen and clomiphene strongly suppressed the infection of P.1.351 and P.1.617 pseudovirus (Fig. 1h, i). Toremifene, the analog of tamoxifen, was reported to treat COVID-19 by blocking S protein.^1^ We further explored whether the binding between S protein and the receptor of SASR-CoV-2, angiotensin-converting enzyme 2 (ACE2), was affected under the treatment of tamoxifen and clomiphene. The binding activity measured by enzyme-linked immunosorbent assay (ELISA) showed that tamoxifen and clomiphene did not affect the binding between the receptor-binding domain (RBD) of SASR-CoV-2 and ACE2 proteins (Fig. 1f). Subsequently, we also tested the inhibition of tamoxifen and clomiphene against SARS-CoV-2 S protein-induced membrane fusion, the result showed that tamoxifen and clomiphene could suppress the membrane fusion which is an important early step for SARS-CoV-2 infection (Fig. 1j). Our study, therefore, suggested that tamoxifen and clomiphene inhibited the infection of SARS-CoV-2 and its variants in vitro through suppressing viral entry on the post-binding stage.

We further examined the inhibition of tamoxifen and clomiphene in mice. The SARS-CoV-2 mouse-adapted strain was used in our study as previously described.^2^ 6-8-week-old BALB/c mice were treated by intraperitoneal injection of tamoxifen and clomiphene prior to infection intranasally with the SARS-CoV-2 mouse-adapted strain. The drug administrations were continued once daily until 3 days post infection (dpi), when the trachea and lung tissues of mice were collected for viral RNA loads assay. The results of the qRT-PCR assay showed that tamoxifen and clomiphene inhibited SARS-CoV-2 RNA loads in the trachea and lung (Fig. 1k, l). In consistent with the qRT-PCR results, we also carried out the SARS-CoV-2 genome RNA in situ hybridization (ISH) assay with RNAscope Reagent Kit. As shown in Fig. 1m, the SARS-CoV-2 genome RNAs while intensively accumulated on the lung blood vessel wall of SARS-CoV-2-infected mice were strongly inhibited in mice treated with either tamoxifen or clomiphene. Finally, we examined the lung inflammation of SARS-CoV-2-infected mice and found that treatment of mice with either tamoxifen or clomiphene strongly decreased the mRNA levels of cytokines such as *TNF-α* and *IL-6* and chemokines such as *CXCL15* and *MCP-1*, in the lung of SARS-CoV-2-infected mice as measured by qRT-PCR (Supplementary Fig. S4a-d). The result of hematoxylin and eosin (H&E) staining of mice lung also showed that both drug treatments strongly reduced the lung tissue inflammation in the lung of SARS-CoV-2-infected mice (Fig. 1n). Above all, our study suggested that tamoxifen and clomiphene inhibited SARS-CoV-2 infection and reduced SARS-CoV-2-induced inflammatory response in mice.

The outbreak of emerging infectious diseases presents huge global challenge to develop vaccines and drugs within very limited time period to control pandemic. In the case of COVID-19 pandemic, SARS-CoV-2 vaccines, such as inactive vaccine, mRNA vaccine, and adenovirus vector vaccine, have been approved in unprecedented speed to be used in people.^3–5^ However, it is unlikely that vaccine along would be able to control this COVID-19 pandemic as multiple dominant SARS-CoV-2 mutant variants have been emerged, some of which can escape from antibody treatment or vaccine protection. In our study, we found that tamoxifen and clomiphene effectively suppressed the infection of not only WT but also mutant SARS-CoV-2 variants such as P.1.351 and P.1.617. These studies suggest that tamoxifen and clomiphene can be used as antiviral agents against different SARS-CoV-2 mutant variants. Furthermore, we have used the SARS-CoV-2-infected mouse model to demonstrate the antiviral activity and anti-inflammation activity of tamoxifen and clomiphene in vivo. Further studies are needed to evaluate the pro and con effects of using these FDA-approved drugs as a therapeutic option for treatment or prevention of infection by the currently circulating SARS-CoV-2 and other emerging coronaviruses in the future.

## Supplementary information

Tamoxifen and clomiphene inhibit SARS-CoV-2 supplemenary

## Data Availability

All data are available within the article, supplementary information, or available from the corresponding author upon reasonable request.
