# Supplementary material for: Tamoxifen and clomiphene inhibit SARS-CoV-2 infection by suppressing viral entry
Source: Signal Transduct Target Ther. 2021 Dec 21;6:435. doi: 10.1038/s41392-021-00853-4 (PMC8688909; doi:10.1038/s41392-021-00853-4)
Supplement: Supplementary file 1 — Tamoxifen and clomiphene inhibit SARS-CoV-2 supplemenary [file 41392_2021_853_MOESM1_ESM.docx]

Supplementary Materials for

Tamoxifen and clomiphene inhibit SARS-CoV-2 infection by suppressing viral entry

Shulong Zu^1,2,3,4^, Dan Luo^4^, Lili Li^1,2,3^, Qing Ye^4^, Rui-Ting Li^4^, Yanan Wang^5^, Meiling Gao^1,2,3^, Heng Yang^1,2,3*^, Yong-Qiang Deng^4*^, Genhong Cheng^6*^

^1^ Center for Systems Medicine, Institute of Basic Medical Sciences, Chinese Academy of Medical Sciences and Peking Union Medical College, Beijing, China

^2^ Institute of Systems Medicine, Chinese Academy of Medical Sciences and Peking Union Medical College, Beijing, China

^3^ Suzhou Institute of Systems Medicine, Suzhou, Jiangsu, China

^4^ State Key Laboratory of Pathogen and Biosecurity, Beijing Institute of Microbiology and Epidemiology, Academy of Military Medical Sciences, Beijing, China

^5^ Suzhou Func Biotech Inc, Suzhou, Jiangsu, China

^6^ Department of Microbiology, Immunology and Molecular Genetics, University of California, Los Angeles, California, USA

Correspondence: Heng Yang ([yhmyt@hotmail.com](mailto:yhmyt@hotmail.com)) or Yong-Qiang Deng ([dengyq1977@126.com](mailto:dengyq1977@126.com)) or Genhong Cheng ([gcheng@mednet.ucla.edu](mailto:gcheng@mednet.ucla.edu))

These authors contributed equally: Shulong Zu, Dan Luo, Lili Li and Qing Ye

**This PDF file includes:**

Materials and Methods

Figure S1 to S4

**Materials and Methods**

***Mice and ethics statement***

6-8-week-old female BALB/c mice were purchased from the Vital River Laboratory. All animal experiments were approved by the Animal Experiment Committee of Laboratory Animal Center, Beijing Institute of Microbiology and Epidemiology (approval number: IACUC-DWZX-2020-002).

***Cells, viruses and antiviral drugs***

Human colorectal adenocarcinoma Caco-2 cell (Cat no. HTB-37), African green monkey kidney Vero cell (Cat no. CCL-81), human kidney epithelial 293T cell (Cat no. CRL-3216) and human alveolar epithelial A549 cell (CCL-185) were purchased from American Type Culture Collection (ATCC). Human live cancer Huh7 cell was from National Institutes for Food and Drug Control (NIFDC). All the cell lines were cultured in Dulbecco’s Modified Eagle Medium (DMEM; Gibco Invitrogen) containing 100 units/mL penicillin (Gibco Invitrogen), 50 μg/mL streptomycin (Gibco Invitrogen) and 10 % fetal bovine serum (FBS; Gibco Invitrogen).

SARS-CoV-2 strain BetaCoV/Beijing/IME-BJ01/2020 and SARS-CoV-2 mouse-adapted strain, MASCp6, have been described previously.[^1^](#_ENREF_1) VSV-based SARS-CoV-2 pseudovirus was from NIFDC.[^2^](#_ENREF_2)

Tamoxifen and clomiphene citrate salt using in this experiment were purchased from MedChemExpress (MCE, Cat no. HY-13757A) and Sigma-Aldrich (Sigma, Cat no. C6272)

***Cytotoxicity assay***

The cytotoxicity of tamoxifen and clomiphene on Vero or Caco-2 cells were determined by [3-(4,5-dimethylthiazol-2-yl)-5-(3-carboxymethoxyphenyl)-2-(4-sulfophenyl)-2H-tetrazolium] (MTS) cell proliferation assays (Promega, Cat no. G3582) as described previously.[^3^](#_ENREF_3) Briefly, cells were seeded into the wells of a 96-well microtiter plate for 12hrs, replacing medium with different doses of either compound or with Dimethyl sulfoxide (DMSO) in triplicate. After 4 days incubation, MTS assays were performed according to the manufacturer’s protocols, and the cytotoxic concentration CC_50_ was calculated using a sigmoidal nonlinear regression function to fit the dose-response curve.

***Measurement of viral RNA***

Viral RNA from cell supernatant and mice tissues have been described elsewhere.[^1^](#_ENREF_1) Viral RNA was performed by qRT-PCR using One Step PrimeScript RT-PCR Kit (Takara, Japan) with the following primers and probe: CoV-F3 (5’-TCCTGGTGATTCTTCTTCAGGT-3’); CoV-R3 (5’-TCTGAGAGAGGGTCAAGTGC-3’); and CoV-P3 (5’-FAM-AGCTGCAGCACCAGCTGTCCA-BHQ1-3’).

***The in vitro antiviral activity***

The *in vitro* antiviral activities of tamoxifen and clomiphene on Vero or Caco-2 cells were determined by qRT-PCR assay as described previously.[^1^](#_ENREF_1) Briefly, the cells were pretreated with different doses of drugs or DMSO and then infected with 100 TCID_50_ of SARS-CoV-2 for 1 hr. The virus-drug mixture was removed and cells were further cultured with fresh drug-containing medium. At 48 hpi, the SARS-CoV-2 RNA copies in supernatant was quantified by qRT-PCR. The EC_50_s of tamoxifen and clomiphene on Vero cells were calculated according to the dose-response curves obtained and analyzed using the GraphPad Prism software.

***Western blot analysis***

Protein samples from SARS-CoV-2 infected Vero cells which were pretreated with tamoxifen or clomiphene were separated by electrophoresis through 10% SDS-PAGE and then the gels were transferred onto polyvinylidene difluoride (PVDF) membranes (Millipore). The membranes were blocked at room temperature for 2 hrs, and subsequently probed with mouse anti-coronavirus spike 2 antibody (MP, Cat no. 087204) and mouse anti-GAPDH antibody (CWBIO, Cat no. CW0100M) as primary antibody and the horseradish peroxidase (HRP)-conjugated Goat Anti-Mouse IgG (ZSGB-Bio, Cat no. ZB-5305) as secondary antibody. Finally, the signals were developed using Immobilon Western Chemiluminescent HRP substrate (Sigma, Cat no. WBKLS0500).

***Immunofluorescence staining***

Vero cells were pretreated with different doses of tamoxifen and clomiphene for 12hrs, then either mock infected or infected with SARS-CoV-2. At 48 hpi, the cells were washed twice with phosphate buffered saline (PBS), fixed with 4% paraformaldehyde and permeabilized with 0.5% Triton X-100. Subsequently, the cells were incubated with the primary antibody (Sino Biological, Cat no. 40150-T62) for 1 hr at 37 °C. After washing three times, the cells were subsequently incubated with the secondary antibody (Bioworld Technology, Cat no. BS10017) for 1 hr at 37 °C. The nuclei were stained with 4,6-diamino-2-phenyl indole (DAPI) (Solarbio, Cat no. C0060) for 5 mins at room temperature. The images were taken by fluorescence microscopy.

***RNA sequencing***

Huh7 cells were pretreated with tamoxifen or clomiphene for 12 hrs. Cells were then infected with SARS-CoV-2. Total RNA was extracted at 48 hpi. The concentration and quality of RNA were measured by the NanoDrop 2000 spectrophotometer (NanoDrop technologies, USA) and an RNA LabChIP 6000 Nano kit (Agilent Technologies, USA). RNA-seq (GENEWIZ, Suzhou, China) were performed. After sequencing, perl script was used to filter the original data (Raw Data) to clean reads by removing contaminated reads for adapters and low-quality reads. Clean reads were aligned to the human genome using Hisat2 v2.1.0. The number of reads mapped to each gene in each sample was counted by HTSeq v0.6.0 and TPM (Transcripts Per Kilobase of exon model per Million mapped reads) was then calculated to estimate the expression level of genes in each sample. DESeq2 v1.6.3 was used for differential gene expression analysis. Genes with padj≤0.05 and |Log2FC| > 1 were identified as differentially expressed genes (DEGs). Heatmaps of gene expression levels were constructed using pheatmap package in R (<https://cran.rstudio.com/web/packages/pheatmap/index.html>).

***SARS-CoV-2 pseudovirus neutralization assay***

Pseudovirus neutralization assay for SARS-CoV-2 were basically performed as described before with some modifications.[^2^](#_ENREF_2) Briefly, Huh7 cells were seeded in a 96-well plate for 12 hrs, subsequently the medium with indicated doses of tamoxifen or clomiphene were transferred to Huh7 cells. After 12 hrs. the cells were infected with SARS-CoV-2 pseudovirus at 2×10^4^ TCID_50_ for 24 hrs. The inhibition rate is calculated by comparing the OD value to the negative and positive control wells.

***Binding assay***

ELISA was performed to evaluate the binding activity of RBD of SARS-CoV-2 spike protein and ACE protein under the treatment of tamoxifen and clomiphene. Briefly, polysorb enzyme-linked immunosorbent assay plates were coated with 25ng per well of RBD protein diluted in PBS overnight at 4 °C. Indicated dose of tamoxifen and clomiphene were mixed with ACE2 protein to make the final concentration of ACE2 to be 0.25μg/mL, 50μL of mixture was added into each well for incubating at 37 °C for 1hr. Horseradish peroxidase was added to the well for 1 h at 37 °C, and then the detection was performed using 3,3′,5,5′-tetramethylbenzidine substrate (Promega, USA). The binding activity of RBD protein and ACE2 protein was calculated according to the highest reciprocal dilution of drug to give an optical density (OD) greater than the sum of the background OD plus 0.01 units. SARS-CoV-2 antibody was used as positive control.

***Membrane fusion assay***

The membrane fusion assay was as previously described.[^4^](#_ENREF_4) Briefly, 293T cells were transfected with GFP tagged SARS-CoV-2 S protein expressing plasmid. After 6 hrs, the 293T cells which were transfected with expressing plasmids and Huh7 cells were co-cultured for 24 hrs. The mixture cells were then fixed and stained by DAPI. Three fields were randomly selected in each well to count the number of fused and unfused cells.

***Mouse challenge experiment***

The mouse challenge experiment has been described previously.[^1^](#_ENREF_1) Briefly, 6-8-week-old female BALB/c mice were administrated intraperitoneally with tamoxifen or clomiphene (60mg/kg) or vehicle 24 hrs prior to infection intranasally with 2×10^4^ TCID_50_ of SARS-CoV-2 mouse adapted strain MASCp6. The administration was continued once daily until 3 dpi, when the lung and trachea tissues of mice were collected for viral RNA loads assay.

***Histopathology assay***

For histopathology, mice lungs were collected and fixed in 4% neutral-buffered formaldehyde, embedded in paraffin, sectioned, and stained with hematoxylin and eosin (H&E). Images were captured by microscope.

***RNA ISH assay***

SARS-CoV-2 genome RNA ISH assay was performed with RNAscope® 2.5 HD Reagent Kit (Advanced Cell Diagnostics) according to the manufacturer’s instruction. Briefly, formalin-fixed paraffin-embedded tissue sections were deparaffinized and endogenous peroxidases were quenched with hydrogen peroxide at room temperature. Slides were then boiled in RNAscope Target Retrieval Reagents and incubated for in RNAscope Protease Plus before probe hybridization. Tissues were counterstained with Gill’s hematoxylin and visualized with standard bright-field microscopy.

***Statistical analysis***

All data were analyzed using the GraphPad Prism 7.01 software. Statistical evaluation was performed by Student’s unpaired t test. Data are presented as mean ± SEM. Significance is denoted as follows: ns, no significance; *P ≤ 0.05; **P ≤ 0.01; ***P ≤ 0.001; ****P ≤ 0.0001.

**Reference**

1 Zu, S. et al. 25-Hydroxycholesterol is a potent SARS-CoV-2 inhibitor. *Cell Res.* **30**, 1043-1045 (2020).

2 Nie, J. et al. Establishment and validation of a pseudovirus neutralization assay for SARS-CoV-2. *Emerg Microbes Infect* **9**, 680-686 (2020).

3 Jin, Z. et al. Structure of M(pro) from SARS-CoV-2 and discovery of its inhibitors. *Nature* **582**, 289-293 (2020).

4 Xia, S. et al. Inhibition of SARS-CoV-2 (previously 2019-nCoV) infection by a highly potent pan-coronavirus fusion inhibitor targeting its spike protein that harbors a high capacity to mediate membrane fusion. *Cell Res.* **30**, 343-355 (2020).

**Supplementary figures**


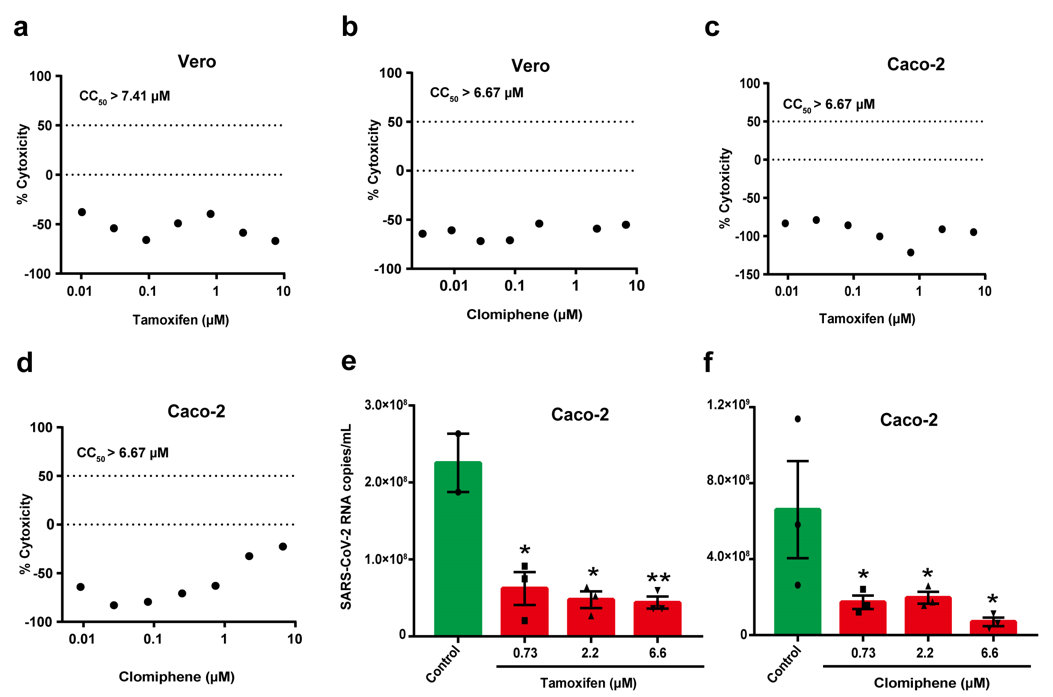
**Supplementary Figure 1.** Tamoxifen and clomiphene were used to calculate CC_50_s on Vero **(a, b)** and Caco-2 **(c, d)** cells; Tamoxifen **(e)** and clomiphene **(f)** inhibit SARS-CoV-2 infection on Caco-2 cell supernatants.


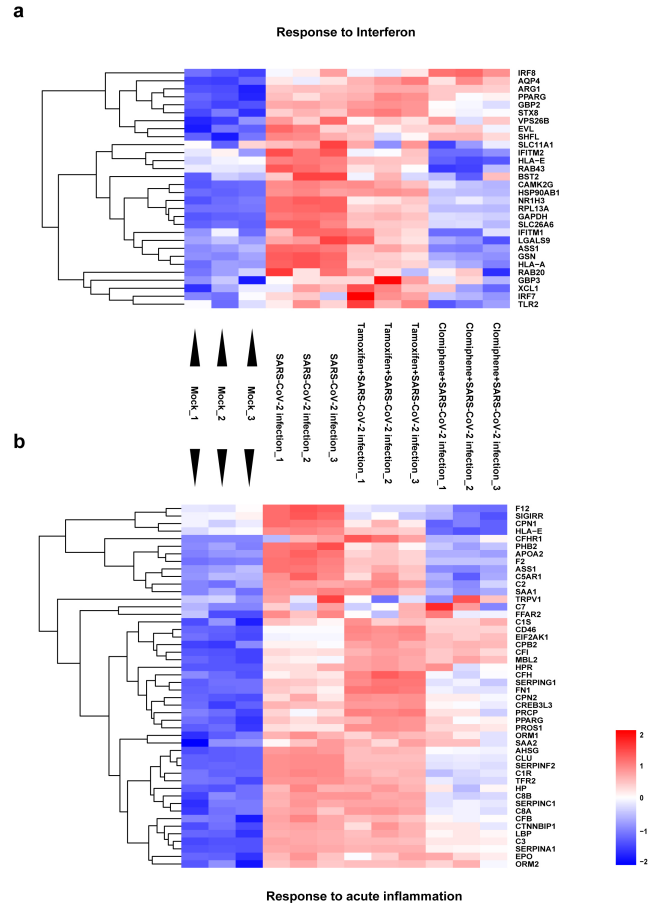
**Supplementary Figure 2.** Tamoxifen and clomiphene suppress SARS-CoV-2 induced interferon **(a)** and acute inflammation responses **(b)**.


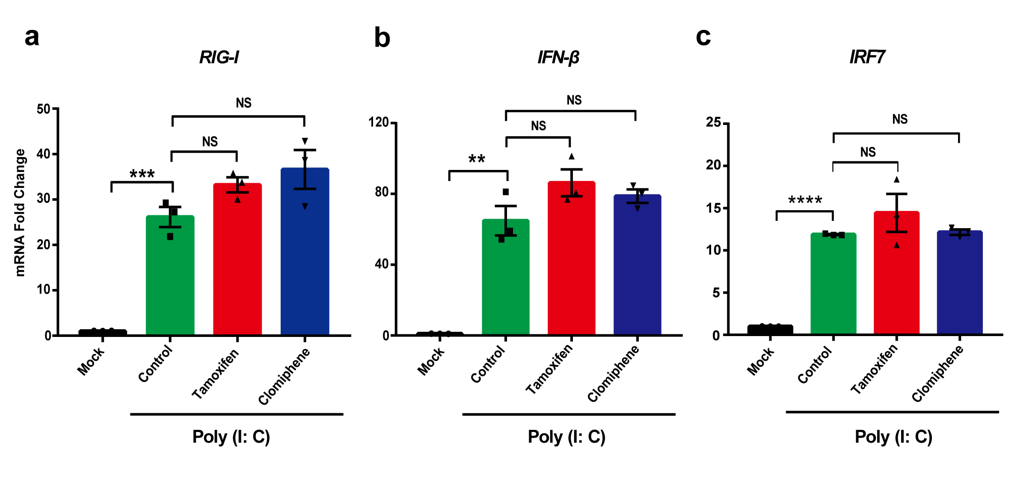
**Supplementary Figure 3.** **(a-c)** A549 cells were pretreated with tamoxifen and clomiphene, then transfected with poly (I:C). After 6 hrs, the cells were collected for testing the mRNA level of indicated genes, including *RIG-I* **(a)**, *IFN-β* **(b)** and *IRF7* **(c)** by qRT-PCR.


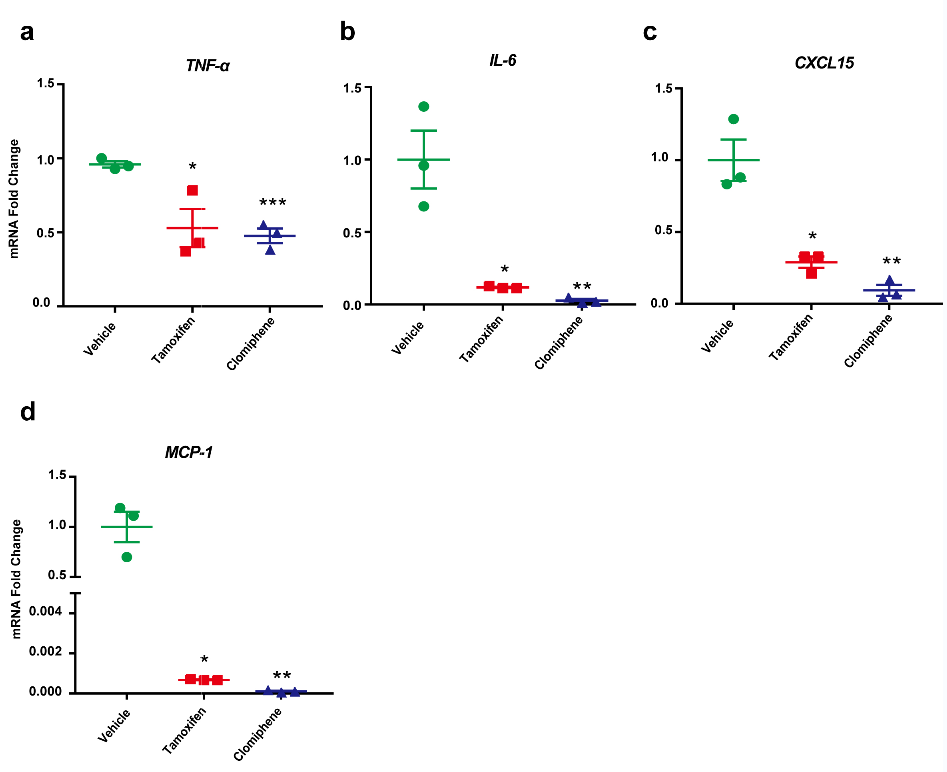
**Supplementary Figure 4.** **(a-d)** Mice lung were used to test the inflammatory cytokine by qRT-PCR, such as *TNF-α* **(a)**, *IL-6* **(b)**, *CXCL15* **(c)** and *MCP1* **(d)**.
